# MG53 Preserves Neuromuscular Junction Integrity and Alleviates ALS Disease Progression

**DOI:** 10.3390/antiox10101522

**Published:** 2021-09-25

**Authors:** Jianxun Yi, Ang Li, Xuejun Li, Kiho Park, Xinyu Zhou, Frank Yi, Yajuan Xiao, Dosuk Yoon, Tao Tan, Lyle W. Ostrow, Jianjie Ma, Jingsong Zhou

**Affiliations:** 1 Department of Kinesiology, College of Nursing and Health Innovation, University of Texas at Arlington, Arlington, TX 76019, USA; jianxun.yi@uta.edu (J.Y.); ang.li3@uta.edu (A.L.); xuejun.li@uta.edu (X.L.); 2 Department of Physiology, Kansas City University of Medicine and Biosciences, Kansas City, MO 64106, USA; marina0207yj@gmail.com (Y.X.); dosuky@gmail.com (D.Y.); 3 Department of Surgery, Davis Heart and Lung Research Institute, The Ohio State University, Columbus, OH 43210, USA; kiho.park@osumc.edu (K.P.); xinyu.zhou@osumc.edu (X.Z.); frank.yi@osumc.edu (F.Y.); tao.tan@osumc.edu (T.T.); 4 Department of Neurology, School of Medicine, Johns Hopkins University, Baltimore, MD 21205, USA; lostrow1@jhmi.edu

**Keywords:** amyotrophic lateral sclerosis, sarcolemma damage, neuromuscular junction, diaphragm, MG53

## Abstract

Respiratory failure from progressive respiratory muscle weakness is the most common cause of death in amyotrophic lateral sclerosis (ALS). Defects in neuromuscular junctions (NMJs) and progressive NMJ loss occur at early stages, thus stabilizing and preserving NMJs represents a potential therapeutic strategy to slow ALS disease progression. Here we demonstrate that NMJ damage is repaired by MG53, an intrinsic muscle protein involved in plasma membrane repair. Compromised diaphragm muscle membrane repair and NMJ integrity are early pathological events in ALS. Diaphragm muscles from ALS mouse models show increased susceptibility to injury and intracellular MG53 aggregation, which is also a hallmark of human muscle samples from ALS patients. We show that systemic administration of recombinant human MG53 protein in ALS mice protects against injury to diaphragm muscle, preserves NMJ integrity, and slows ALS disease progression. As MG53 is present in circulation in rodents and humans under physiological conditions, our findings provide proof-of-concept data supporting MG53 as a potentially safe and effective therapy to mitigate ALS progression.

## 1. Introduction

Amyotrophic lateral sclerosis (ALS) is a fatal neuromuscular disease characterized by progressive motor neuron loss and muscle atrophy [1,2]. Progressive respiratory muscle weakness is a main cause of morbidity and eventual death [3,4]. ALS appears to be a combination of “dying-forward” and/or “dying-back” pathophysiological processes, starting in cortical motor neurons and glia or at the muscle and neuromuscular junction (NMJ) [5,6,7,8,9,10,11,12,13,14,15,16]. It is possible that the balance of these processes differs in subsets of ALS patients, and multiple mechanisms have been proposed for how they may interrelate. Previous studies have shown that NMJ degeneration is associated with mitochondrial dysfunction in ALS [17,18,19,20,21], which involves bidirectional crosstalk between myofibers and neuron [6,8,9,17,18,19,20,21]. Therapeutic approaches that sustain NMJ integrity and muscle function could slow disease progression. Thus, it is critical to understand the molecular mechanisms associated with NMJ degeneration in ALS.

During respiration, the diaphragm constantly undergoes contraction–relaxation, a process that leads to injury to the muscle membrane. Inadequate repair of injury to the sarcolemma can disrupt NMJ integrity and contribute to diaphragm wasting in ALS. MG53, a member of the tripartite motif (TRIM) protein family [22], was identified as an essential component of the cell membrane repair machinery [23,24,25,26]. Genetic ablation of MG53 results in defective membrane repair and tissue regenerative capacity [23,24,27,28]. A series of studies has shown that recombinant human MG53 (rhMG53) protein protects various cell types against membrane disruption when applied to the extracellular environment and ameliorates pathology associated with muscular dystrophy [29], acute lung injury [30], myocardial infarction [31], acute kidney injury [32], and ischemic brain damage [33] in animal models.

In this study, we identify NMJ as an active site of injury repair by MG53 and show that compromised diaphragm muscle membrane repair occurs prior to symptom onset in ALS mice. Diaphragm muscles from ALS mouse models show increased susceptibility to injury and intracellular MG53 aggregation. This pathological feature is also revealed in human muscle samples from ALS patients. Remarkably, systemic application of rhMG53 in ALS mice protects against injury to the diaphragm, preserves integrity of NMJ, and significantly alleviates ALS disease progression.

## 2. Materials and Methods

### 2.1. Animal Models

The ALS transgenic mouse model (G93A) with genetic background of B6SJL was originally generated by Drs. Deng and Siddique’s group at Northwestern University [34], which was also deposited to the Jackson Lab as B6SJL-Tg (SOD1*G93A). We obtained this colony from Dr. Deng and maintained it through breeding the B6SJL/G93A male mice with B6SJL/WT female mice. All experiments were carried out in accordance with the recommendations in the Guide for the Care and Use of Laboratory Animals of the National Institutes of Health. Protocols on the usage of mice were approved by the Institutional Animal Care and Use Committee of the University of Missouri at Kansas City, Kansas City University of Medicine and Biosciences, and University of Texas at Arlington. Sprague-Dawley rats (4-month-old) were purchased from Charles River Laboratories. Protocol on the usage of rats was approved by the Institutional Animal Care and Use Committee of the Ohio State University.

### 2.2. Isolation of Single Live FDB Muscle Fibers (Myofibers)

Experimental mice were euthanized by cervical dislocation, and flexor digitorum brevis (FDB) muscles were removed for enzyme digestion to obtain individual myofibers for functional or biochemical studies [18,20,21]. Briefly, FDB muscles were digested in modified Krebs solution (0 Ca^2+^) containing 0.2% type I Collagenase (Sigma-Aldrich, St. Louis, MO, USA) for 1 h at 37 °C. After digestion, muscles were kept in collagenase-free Krebs solution (with 2.5 mM Ca^2+^ and 10 mM glucose) at 4 °C and used for studies within 24 h.

### 2.3. Confocal Imaging and Image Analysis

A Leica TCS SP8 confocal microscope (Leica Microsystems Inc., Wetzlar, Germany) was used for imaging. Images were captured with either 40×, 1.2 NA water immersion objective or 63×, 1.4 NA oil immersion objective. Imaging was conducted at room temperature (~23 °C). Background correction, binary segmentation, and polyline kymograph analysis (for fluorescent intensity profiling over distance) were performed using Fiji (ImageJ).

### 2.4. Imaging of T-Tubule Network Integrity and Mitochondrial Membrane Potential at NMJ

Freshly isolated FDB myofibers were seeded in laminin (Santa Cruz sc-29012)-coated glass bottom dishes and stained with TMRE (1: 3000 dilution from 150 μM stock solution, Invitrogen T669), BTX–Alexa Fluor 488 (1:1000 dilution from 1 mg/mL stock solution, Invitrogen B13422), and CellMask DeepRed (1:1000 dilution from 5 mg/mL stock solution, Invitrogen C10046) for 30 min. The myofibers were washed 5 times with 2.5 mM Ca^2+^ Krebs solution before imaging with confocal microscopy. BTX–Alexa Fluor 488 was excited by 488 nm laser (emission filter 500–550 nm). TMRE was excited by 568 nm laser (emission filter 575–625 nm). CellMask DeepRed was excited by 633 nm laser (emission filter 640–700 nm).

### 2.5. Evaluation of Mitochondrial ROS Level in Live FDB Myofibers

The fluorescent dye MitoSOX^TM^ Red (M36008, Invitrogen, Waltham, MA USA) was used to evaluate mitochondrial superoxide level in live FDB myofibers. FDB myofibers were incubated with 1 µM MitoSOX^TM^ Red in Krebs solution for 10 min at 37 °C. MitoSOX^TM^ Red was excited by 514 nm laser (emission filter 570–600 nm). All parameters for imaging collection were kept the same between control and experimental groups, which included the power of the laser used, the pinhole, and the gain of the fluorescence recording.

### 2.6. Evaluation of Membrane Repair Function in FDB Myofibers

We adapted a laser-induced membrane damage protocol from our early publications [23,29] to evaluate the membrane repair function of live myofibers. An amount of 2 μM of FM 1–43 fluorescent dye was added to the medium of freshly isolated FDB myofibers [23,26] with 50 µM n-benzyl-p-toluene sulfonamide to prevent myofiber contraction [20,35]. Using the FRAP protocol on the Leica TCS SP8 confocal microscope, a small area of the myofiber (12 μm × 12 μm) was exposed to a high intensity laser (488 nm, 50% power) to cause a localized muscle membrane injury. The time-dependent accumulation of FM 1–43 fluorescent signal inside the myofiber after the laser-induced membrane injury was recorded every 10 s for 5 min. FM 1–43 was excited by 488 nm laser (emission filter 550–610 nm). All parameters for imaging collection were kept the same for all tested myofibers, which included the power of the laser used, the pinhole, and the gain of the fluorescence detector. The individual data point of the fluorescent intensity was calculated as (F − F_0_), in which the background fluorescence (F_0_) was corrected for each data point.

### 2.7. Quantification of Membrane Integrity of Diaphragm Muscle

EB dye (1% in PBS, 10 μL/g body weight) was applied to mice via intraperitoneal (IP) injection [29]. Sixteen hours later, diaphragm muscle was removed from mice and immediately immersed in Krebs solution for evaluation under a confocal microscope of the EB fluorescence intensity retained inside myofibers. EB was excited by 633 nm laser (emission filter 640–700 nm). All parameters for imaging collection were kept the same between control and experimental groups, which included the power of the laser used, the pinhole, and the gain of the fluorescence recording.

### 2.8. Immunohistochemistry of Mouse and Human Muscle

For whole-mount fixed muscle assay, the intact diaphragm, tibialis anterior (TA), extensor digitorum longus (EDL), and soleus muscles were dissected from the experimental mice and fixed in either methanol (precooled at −20 °C) for 10 min or 4% paraformaldehyde (PFA) overnight at 4 °C. For PFA fixed samples, the reaction was stopped by PBS containing 1% glycine. The samples were then washed with PBS, dehydrated, and rehydrated through a graded series of alcohol. Pre-blocking was performed at room temperature for 2 h in blocking buffer containing 3% goat serum, 3% BSA, 0.1% Tween–20, 0.1%, Triton X–100 and 0.1% NaN_3_. The whole-mount fixed muscle samples were then incubated with primary antibody at 4 °C overnight (anti-neurofilament: Abcam, ab8135 1:250 dilution; anti-Mg53 antibody: 1:200 dilution [32,36,37,38]). After PBS washing, the samples were incubated for 4 h with Alexa Fluor conjugated secondary antibodies of the corresponding species/isotype (1:1000). The samples were then washed again, cleared in glycerol, and mounted in anti-fade mounting medium (Tris buffer containing 60% glycerol and 0.5% N– propyl gallate) for imaging under confocal microscope. For the NMJ staining, BTX–Alexa Fluor 488 (1:1000 dilution) was added during the second antibodies’ incubation.

Formalin-fixed, paraffin-embedded slides of postmortem ALS and non-neurological control decedent muscle were obtained from the Target ALS Multicenter Postmortem Tissue Core. All decedents underwent standard autopsies with consents for autopsy obtained from next-of-kin after death and HIPAA Form 5 exemptions to access and share de-identified clinical data from decedents. Slides were provided blinded to whether they were from ALS or control autopsies and were then unblinded after staining and analysis was completed.

For paraffin sectioning, muscle samples were fixed in 4% paraformaldehyde (PFA) overnight at 4 °C, washed with PBS, and dehydrated through a graded series of alcohol. Clearance was done with xylene, and paraffin embedding was carried out under negative pressure for 3 h. Samples were cut into 8 μm sections for immunostaining. Antigen retrieval was carried out at 95 °C for 20 min in citrate buffer (pH 6.0).

For dissociated FDB myofibers, the samples were allowed to attach to laminin (sc-29012)-coated glass bottom dishes for 30 min in culture medium containing Alexa Fluor-coupled BTX (1:1000) before fixation (4% PFA for 15 min at 37 °C). The pre-blocking, primary, and secondary antibody incubation steps were the same as described above.

### 2.9. Immunoblotting Assay

The mouse serum samples (1.5 µL each) were resolved in 8.7% SDS polyacrylamide gels and transferred to a polyvinylidene difluoride (PVDF) membrane and probed with an anti-MG53 antibody 1:2000. Ponceau S staining was used to verify equal loading of the serum samples. Proteins from mouse TA muscle were extracted with RIPA/protease inhibitors and resolved by 10% SDS–PAGE, then transferred to PVDF membrane and probed with antibodies against MG53 antibody 1:5000 and GAPDH (1:10,000, from CST). Protein bands were visualized with ECL reagents under ChemiDoc Imaging system (Bio-Rad Laboratory). Band intensity was analyzed with ImageJ software (NIH, Bethesda, MD, USA).

### 2.10. Evaluation of Serum Creatine Kinase (CK) Activity

Blood samples (~100 μL/mouse) were collected from the tail vein of mice at rest. Then the same group of mice were subjected to a 30 min running protocol at 18 m/min, 15° downhill. Right after completion of the running protocol, the blood samples were collected again. The serum was collected from supernatant after centrifuging the blood samples at 2000 *g* for 10 min at 4 °C. Freshly made serum samples were used to measure CK activity following the protocol provided by Sigma (MAK–116). Briefly, for each reaction, 10 μL of serum was mixed with 100 μL assay buffer; the mixture was then loaded onto a 96-well plate for recording the absorbance at 340 nm at time intervals of 5 min up to 40 min in a plate reader (SpectraMax i3x). The CK activity was calculated according to the formula provided:

CK Activity (units/L) = ((A_340nm_)_(at 40 min)_ − (A_340nm_)_(at 5 min)_/(A_340nm_)_calibrator_ − (A_340nm_)_blank_) × 150

### 2.11. Quantification of Motor Neurons in Lumbar Spinal Cord with Nissl Staining

The lumbar portion of the spinal cord was collected from the mice and fixed in 3.7% PFA at 4 °C overnight and stored in 70% ethanol until use. After embedding in 6% LMP agarose, the fixed spinal cords were cut in 25 µm sections starting at the upper part to subsequently collect 20 sections from each lumbar spinal cord using a Vibratome (Leica VT1000S, Nussloch, Germany). The spinal cord sections were stained with 0.1% Cresyl violet acetate. The motor neurons (Nissl substrate-positive with diameter larger than 25 µm) in the anterior horn region of each section were quantified in a double-blind manner for different treatment groups.

### 2.12. PEGylation of rhMG53

PEGylation is a well-established method for increasing the circulating half-life of therapeutic proteins [39]. We conducted a study with mPEGSVA (purchased from LaySan Bio, Inc., Arab, AL, USA) modification of rhMG53. An amount of 40 mg rhMG53 protein was dissolved in PBS (pH = 8) solution at 1 mg/mL concentration at 4 °C. Then, 40 mg PEG-SVA was added into the rhMG53 solution and mixed gently. The reaction of PEGylation was carried out at 4 °C overnight. Un-conjugated PEG–SVA was filtered out by Amicon 30 ultrafilter with PBS. PEG–rhMG53 was stored at −20 °C for long-term storage or 4 °C for short-term usage and to avoid the freeze–thaw cycle.

### 2.13. Pharmacokinetic Evaluation of PEG–rhMG53 in Rats

Rats were anesthetized by isoflurane inhalation. rhMG53 or PEG–rhMG53 were injected intravenously via tail vein. Blood samples were collected from the tail vein at 15 min, 30 min, 1 h, 2 h, 6 h, 12 h, 24 h, 48 h (rhMG53 only), and 96 h (PEG–rhMG53 only). Serum was collected by centrifugation of clogged blood at 8000 rpm at 4 °C for 10 min and diluted at 1:50 for ELISA assay to quantify the serum levels of rhMG53. Specifically, the ELISA plate (Nunc-Immuno™ plates, 96-well-plate, MaxiSorp) was coated 100 uL/well with an anti-MG53 rabbit monoclonal antibody (10 μg/mL) in coating buffer (Na_2_CO_3_: 3.18 g; NaHCO_3_ 5.88 g to 1000 mL, pH = 9.6) at 4 °C, overnight. ELISA washing and blocking reagent were purchased from KPL. Diluted serum samples as well as rhMG53 standards were added into the coated ELISA plates and incubated for 1.5 h. After 4X washing, biotinylated anti-MG53 5259 (1:500, 100 μL/well) was added as the detection antibody and incubated for 1.5 h. After 4X washing, HRP-Streptavidin (HRP-Conjugated Streptavidin (Thermo Scientific, Waltham, USA; Cat No: N100), 1:5000 dilute in blocking buffer) was added and incubated for 30 min at room temperature. After 5X washing, KPL SureBlue Reserve^TM^ TMB microwell peroxidase substrate (Cat No: 53-00-02) was added and incubated till blue color developed, then read O.D. at 650 nm. Note that the reason we used rats instead of mice for the pharmacokinetic evaluation of PEG–rhMG53 is because rats have much more total blood volume (25 mL) than mice (1.5 mL). Thus, the multiple blood collections would have a minimal effect on rats.

### 2.14. Intravenous Administration of rhMG53 and PEG–rhMG53

Stock solution of rhMG53 or PEG–rhMG53 was prepared by dissolving rhMG53 or PEG–rhMG53 in sterilized saline solution at the concentration of 2 mg/mL. A 3/10 cc insulin syringe with gauge 31 needle was used for the injection. The experimental G93A mouse received isoflurane inhalation (1–2%) to reach an appropriate anesthesia state, at which time the mouse showed no response to pinching at their toes. Then, the submandibular vein area of the mouse was cleaned with 70% alcohol wipe, and then rhMG53 or PEG–rhMG53 solution (~30 µL) was injected into the submandibular vein. The G93A mice in the control group received same amount of saline injection. The isoflurane inhalation was immediately stopped after the injection. Usually, the mouse resumed normal activities within five minutes after stopping isoflurane inhalation. The submandibular vein on both sides was used alternatively for the intravenous administration.

### 2.15. Numerical Data Presentation and Statistics

All measurements were taken from distinct samples. Data are presented as mean ± S.E. Statistical comparisons were done using Students’ *t*-test (two-sided) for single mean or ANOVA test for multiple means when appropriate (we assumed data to be normally distributed). Pearson’s chi-squared tests were carried out by RStudio. Pearson’s correlation coefficient for colocalization analysis was calculated by Coloc 2 plugin in ImageJ following the equation below:

$$r=\left[n\left(\sum^{\;}xy\right)-\left(\sum^{\;}x\right)\left(\sum^{\;}y\right)\right]/\sqrt{[n\sum^{\;}x^{2}-\left(\sum^{\;}x{)}^{2}\right][n\sum^{\;}y^{2}-\left(\sum^{\;}y{)}^{2}\right]}$$

Line plots were generated in either Sigmaplot (Systat Software Inc., Palo Alto, CA, USA) or Excel. Box-and-dot plots were created by the ggplot2 package of RStudio. The box bottom, median line, and box top represent the 25th (Q1), 50th (Q2), and 75th percentile (Q3), respectively. Whisker ends represent Q1 − 1.5 * IQR and Q3 + 1.5 * IQR. IQR is interquartile range (Q3–Q1). *p* < 0.05 was considered statistically significant.

## 3. Results

### 3.1. Increased Susceptibility to Diaphragm Injury Is an Early Pathology Event in SOD1(G93A) Mice

**Figure 1:**
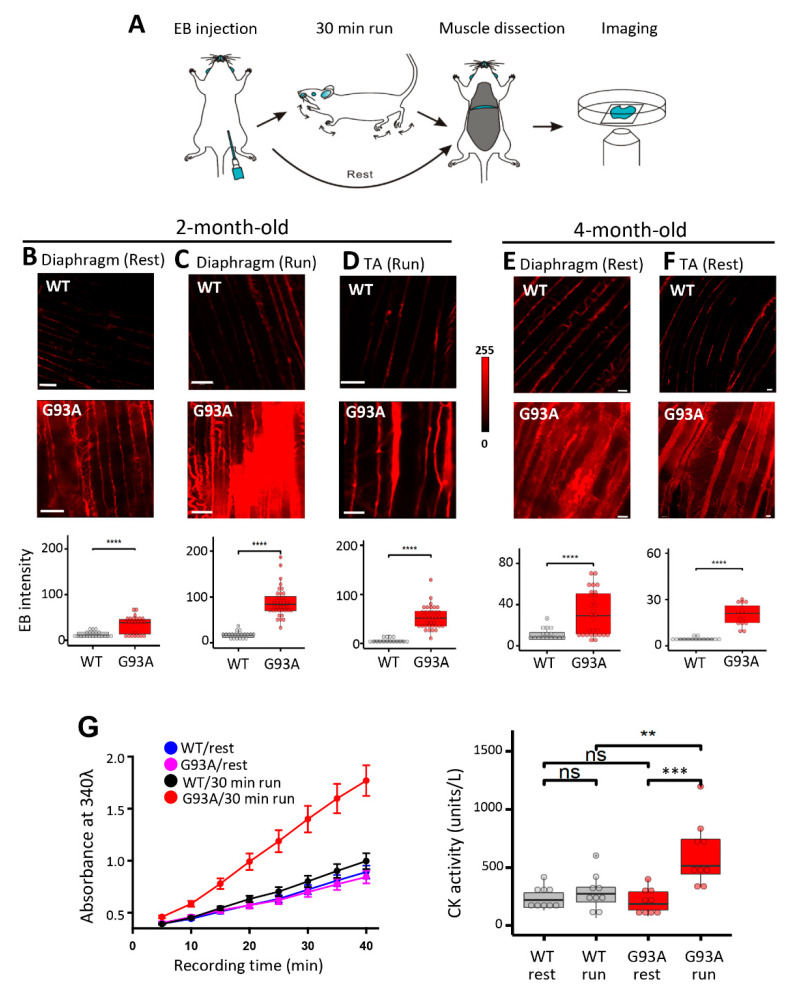
Diaphragm susceptibility to membrane injury is an early pathological event in ALS. (**A**) Schematic diagram for evaluation of diaphragm integrity in ALS mice. (**B**) Compared with WT mice, diaphragm muscles derived from G93A littermates (2-month-old) showed increased EB intensity at rest (WT: 12.8 ± 1.5, *n* = 21 from 4 mice; G93A: 33.4 ± 3.7, *n* = 26 from 2 mice, **** *p* < 0.0001). Scale bars: 20 µm. (**C**) 30 min downhill running caused drastic elevation of EB in diaphragm derived from the G93A mice (WT17.30 ± 1.69, *n* = 20 from 3 mice; G93A: 88.49 ± 4.92, *n* = 39 from 4 mice, **** *p* < 0.0001). (**D**) 30 min downhill running also caused elevation of EB accumulation in G93A TA muscle (WT: 5.37 ± 1.13, *n* = 16 from 2 mice vs. G93A: 52.40 ± 3.83, *n* = 36 from 3 mice, **** *p* < 0.0001). (**E**) Evaluation of EB accumulation in G93A diaphragm muscle of 4-month-old G93A mice at rest (G93A: 31.1 ± 4.0, *n* = 29 from 3 mice vs WT: 10.9 ± 1.6, *n* = 16 from 2 mice, *** *p* < 0.001). Scale bars: 20 µm. (**F**) Evaluation of EB accumulation in TA muscle of 4-month-old G93A mice at rest (WT: 4.46 ± 0.27, *n* = 15 from 2 mice vs G93A: 20.18 ± 1.77, *n* = 15 from 3 mice, **** *p* < 0.0001). (**G**) Quantification of CK activity in serum samples of G93A and WT littermate mice (2-month-old) before and after 30 min running. *n* = 9 for each group, ** *p* < 0.01, *** *p* < 0.001, ns: not significant. Left panel shows the time-dependent reading of the absorbance (at 340 nm) of serum samples in a 96-well plate; Right panel shows the calculated CK activity derived from the absorbance reading (see Materials and Methods section). Scale bars: 20 µm.

Transgenic mice expressing the human SOD1*^G93A^* mutation (G93A) are a highly characterized and widely used animal model for preclinical investigation of pathogenic mechanisms in ALS. The particular G93A ALS mouse model we used develop ALS symptoms at the age of ~3 months old and have a life span of ~4 to 5 months [40,41]. As illustrated in Figure 1A, we performed intraperitoneal (IP) injections of Evans blue (EB) dye to wild-type (WT) and G93A littermates at 2 months of age (prior to ALS onset) [34]. We harvested the diaphragm muscles 16 h later, immediately after the mice performed a 30 min running protocol (18 m/min, 15° downhill), and evaluated EB retention within muscle myofibers, which measures the extent of muscle membrane leakage [42]. The G93A diaphragm muscles displayed significantly higher EB levels under resting condition compared with WT (Figure 1B). The 30 min downhill protocol further exacerbated EB accumulation in the G93A diaphragm, whereas no significant changes were measured in the WT diaphragm (Figure 1C). The same exercise-induced excessive membrane damage was also observed in the tibialis anterior (TA) muscle derived from the G93A mice (Figure 1D).

To determine whether this muscle membrane damage could be detected systemically, we measured the serum creating kinase (CK) level. The 2-month-old G93A and WT mice were subjected to the same 30 min running protocol, and the blood samples were collected from the mice before and after the running protocol. Measurement of CK showed significant elevation in 2-month-old G93A mice after running (Figure 1G). These data indicate that ALS muscle, especially the diaphragm muscle, exhibits membrane damage, which occurs before clinical ALS symptom onset and can be further exacerbated by muscle contraction activity. Enhanced EB retention in the diaphragm (Figure 1E) and TA muscles (Figure 1F) was also revealed after the ALS symptom onset in 4-month-old G93A mice without running, indicating that muscle injury and fragility continue during ALS disease progression.

### 3.2. MG53 Is Implicated in the Membrane Fragility of G93A Diaphragm, Fast- and Slow-Type Muscles

**Figure 2:**
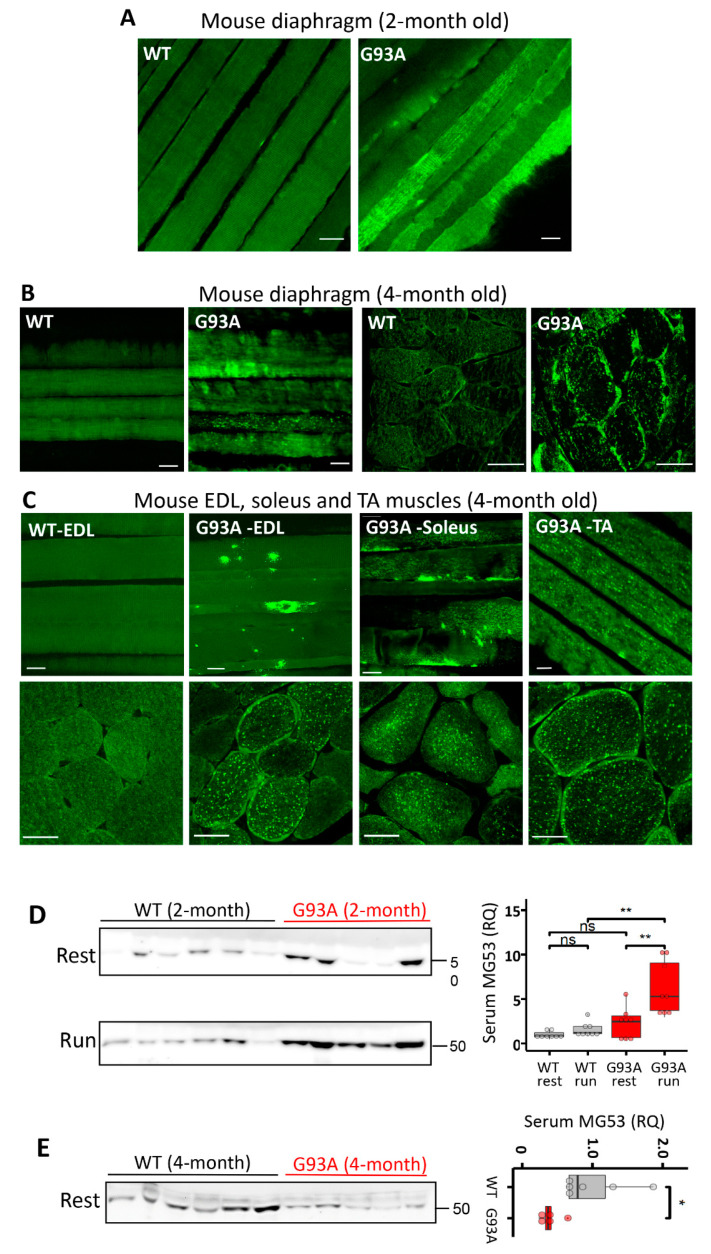
MG53 is implicated in the membrane fragility of G93A diaphragm, fast- and slow-type muscles. (**A**) MG53 immunostaining of diaphragm muscle of WT and G93A mice after the 30 min running (2-month-old, before ALS onset). The whole-mount longitudinal views revealed homogenous patterns of MG53 in WT diaphragm, whereas the G93A diaphragm showed aggregation and patching patterns of MG53 after running, indicative of membrane injury. Scale bars: 20 µm. (**B**) MG53 immunostaining of diaphragm muscle of WT and G93A mice at rest (4-month-old, after ALS onset) revealed intracellular aggregates of MG53 in G93A diaphragm. Scale bars: 20 µm. (**C**) MG53 immunostaining of skeletal muscles isolated from multiple anatomic locations in 4-month-old WT and G93A mice in cross sections and whole-mount preparations. There were prominent intracellular MG53 protein aggregates in all G93A muscles examined, while there was no apparent MG53 aggregation inside the WT muscles. Scale bars: 20 µm. (**D**) Immunoblotting of serum MG53 levels in WT and G93A littermate mice (2-month-old) before (rest) and after 30 min running (run). Ponceau S staining indicated equal loading of the serum samples (see Supplementary Figure S1). WT mice showed no significant changes in the serum MG53 level before and after running (WT/rest: 1.00 ± 0.14 vs. WT/run: 1.58 ± 0.28, ns: *p* > 0.05). After running, G93A mice showed a significant increase in serum MG53 level (G93A/rest 2.29 ± 0.63 vs. G93A/run: 6.21 ± 1.09, ** *p* < 0.01). *n* = 8/group. (**E**) G93A mice at the age of 4 months without running showed significantly lower levels of serum MG53 compared with WT littermates (G93A, *n* = 5; WT, *n* = 6, * *p* < 0.05).

Multiple key proteins form essential components of the cell membrane repair. Among them MG53 is the first to arrive at the injury site (within 2 s) to assemble a repair patch at the membrane injury site [43]. We performed MG53 immunostaining in diaphragms derived from WT and G93A mice at the age of two months, immediately after running. The whole-mount preparation (longitudinal view) (Figure 2A) revealed distinct membrane localized patterns of MG53 in the G93A diaphragm, which were rarely seen in the WT diaphragm. This observation is consistent with the notion that G93A diaphragm muscles are susceptible to exercise-induced injury, and endogenous MG53 translocates to the areas of membrane damage in ALS muscle, consistent with its known functions in promoting plasma membrane repair.

We next examined the expression pattern of MG53 in the diaphragm of G93A mice at a later ALS stage (4-month-old). As shown in Figure 2B, in sharp contrast with the WT diaphragm muscle, both longitudinal (whole-mount preparation) and transverse sections of G93A diaphragm showed abnormal intracellular MG53 aggregates. The intracellular MG53 aggregation was observed in all examined muscles derived from late-stage G93A mice (4-month-old), including extensor digitorum longus (EDL), soleus, and tibialis anterior (TA) (Figure 2C). The cytosolic aggregation may compromise MG53′s membrane repair function in G93A skeletal muscle.

MG53 protein can be released from skeletal muscle as a myokine [44]. In *mdx* mice, a model of Duchenne muscular dystrophy, we previously demonstrated that increased serum MG53 level correlated with an increased serum CK level [29], indicating that elevated serum MG53 level serves as a biomarker for muscle membrane damage. Blood samples were collected before and after 30 min running of 2-month-old G93A and WT mice (as in Figure 1) to quantify MG53 levels in the serum. As shown in Figure 2D, the 30 min running resulted in a significant increase in the serum MG53 level in G93A mice, but had no impact on WT mice. These findings further support that skeletal muscle membrane injury represents an early pathological event in ALS G93A mice. We further compared the MG53 expression level in skeletal muscle of G93A and WT mice at the age of 2 and 4 months. As demonstrated in Supplementary Figure S2, there is a significant increase in MG53 level in G93A mice at the age of 4 months after ALS onset, which is in line with the increased MG53 aggregates detected in G93A muscle after the disease onset. Interestingly, the serum level of MG53 in the 4-month-old G93A mice at late stage of ALS was lower compared with the WT littermates under resting conditions (Figure 2E). As MG53 is a muscle-derived protein, the reduced serum MG53 level could either be due to the progressive loss of muscle mass or the exacerbated intracellular aggregation of MG53, which sequesters and hence limits its release into circulation. The increased MG53 level in later stage G93A muscle could reflect a compensatory reaction to the muscle injury or the reduced degradation of aggregated protein during ALS progression. However, the endogenous MG53 can no longer sustain the membrane repair function as ALS progresses.

### 3.3. NMJ Is an Active Site of Injury Repair by MG53 that Is Lost in ALS

**Figure 3:**
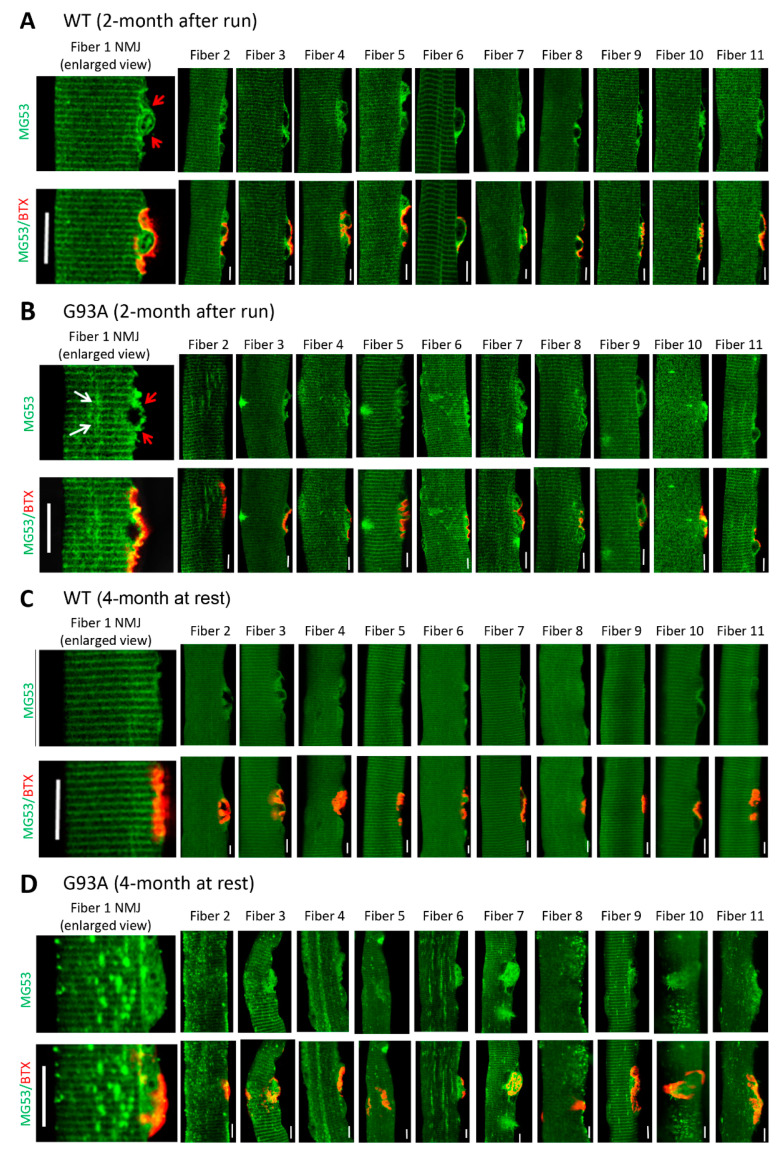
MG53 forms membrane repair patches at NMJ under physiological condition and aggregates near NMJ in G93A myofibers. (**A**) FDB myofibers derived from WT mice (2-month-old) subjected to 30 min running were co-stained with MG53 antibody (green) and BTX (red). MG53 formed membrane patches covering the NMJ site (indicated by red arrows in the enlarged images of NMJ on the left most panel). More representative images of myofibers confirm this observation (right panels). (**B**) FDB myofibers derived from G93A WT mice (2-month-old, before ALS onset) subjected to 30 min running. Similar to the WT myofibers, MG53 also formed membrane patches covering the NMJ site. However, intracellular MG53 aggregates start to appear near the site of NMJ. (**C**) FDB myofibers derived from WT mice (4-month-old, without running) show a uniform pattern of MG53. (**D**) In sharp contrast with the WT myofibers, the myofibers derived from G93A mice at the advanced stage of ALS (4-month-old, without running) displayed extensive MG53 aggregates near NMJ without running. Scale bars: 20 µm.

We showed previously that segmented mitochondrial defects appeared locally at NMJs in the G93A mice [20,21]. This prompted us to examine whether NMJ itself is an active site of membrane injury and whether MG53 plays a role in repair of NMJ injury. Due to its shorter myofiber length, the flexor digitorum brevis muscle (FDB) can be enzyme-digested to yield live single myofibers for morphological and functional assessments. Immediately after the 30 min running, freshly isolated FDB myofibers from 2-month-old mice were fixed for staining with α-Bungarotoxin (BTX) to mark NMJ and anti-MG53 antibodies. We found that MG53 accumulated at the NMJ area and formed patches in WT myofibers (*n* = 130, four mice) (Figure 3A, left panel) with more representative images presented in the right panel, suggesting that MG53 contributes to the maintenance of NMJ integrity under physiologic conditions. While MG53 also accumulated at the NMJ area in G93A myofibers (*n* = 109, three mice), the patches were sparse and often not uniform in shape and size in about 10% of G93A myofibers (Figure 3B).

FDB myofibers derived from G93A mice at 4 months old showed abnormal MG53 aggregation near NMJs even without running (Figure 3D), which is in sharp contrast with the uniform pattern of MG53 distribution near NMJs in WT myofibers (Figure 3C). Such MG53 aggregation suggests potential impaired tissue repair capacity that may manifest in NMJ degeneration during ALS disease progression. Note that the MG53 aggregates not only appear near NMJ but also spread to the entire myofiber, similar to the phenomenon observed in diaphragm, EDL, TA, and soleus muscles (Figure 2B,C).

**Figure 4:**
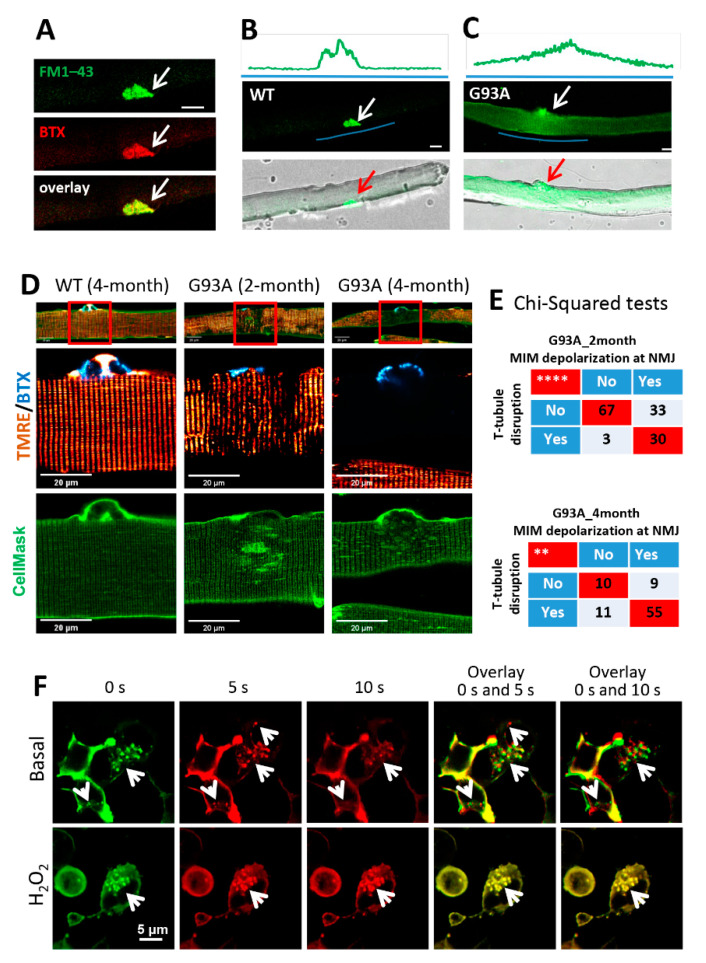
NMJ is more susceptible to injury than other regions of the sarcolemma, and mitochondrial dysfunction could underlie the impairment of the MG53-mediated membrane repair mechanism in G93A myofibers. (**A**) FM 1–43 and BTX co-localized at the NMJ of FDB myofibers (white arrows). (**B**) No intracellular FM 1–43 fluorescence was observed in the WT myofiber at 20 min after incubation with FM 1–43. (**C**) FM 1–43 entered the G93A myofiber via the NMJ region. Green line highlights the region for fluorescence intensity profiling. Scale bars: 20 µm. (**D**) FDB myofibers were loaded with BTX (cyan), TMRE (red), and CellMask (green) simultaneously. At the site of NMJ, mitochondria depolarization (loss of TMRE fluorescence) occurred in myofibers derived from both 2-month-old and 4-month-old G93A mice, accompanied by disrupted T-tubule network (marked by CellMask). Normal mitochondria and organized T-tubule network were detected at the site of NMJ of WT myofiber. Scale bar: 20 µm. (**E**) Chi-square tests indicate positive correlations between mitochondrial inner membrane (MIM) depolarization and T-tubule disorganization at the site of NMJ of G93A mice at both 2 months and 4 months of age (** *p* < 0.01, **** *p* < 0.0001, 3 mice/group). (**F**) The representative time-lapse images of C2C12 cells with overexpression of GFP–MG53 in the presence and absence (basal) of 1 mM H_2_O_2_. The overlay images were generated using images at 0 s and 5 s, or 0 s and 10 s. White arrows indicate GFP–MG53 vesicles. Scale bars: 5 µm.

Using the entry of a cell-impermeable fluorescent dye FM 1–43 as a measure of muscle myofiber integrity [23,45], we tested the possibility that ALS-associated muscle injury originates focally at NMJs. We used α-BTX to label NMJ in the FDB fiber. Figure 4A shows an overlay of FM 1–43 and α-BTX, indicating FM 1–43 enriches at the NMJ first when applied to the medium. In WT myofibers, FM 1–43 was restricted to the NMJ, and there was barely any FM 1–43 entry inside myofibers after a 20 min incubation (Figure 4B). In contrast, 59% ± 7% of FDB myofibers from three 4-month-old G93A mice showed intracellular uptake of FM 1–43 dye, which formed a gradient centered around the NMJ (Figure 4C), indicating that FM 1–43 dye entered the cells preferentially through the injured NMJs.

We next examined whether exercise could exacerbate membrane injury at NMJs of G93A muscle. For this purpose, three pairs of 2-month-old WT and G93A littermate mice performed the running protocol for 30 min. A portion of 72% ± 2% of G93A FDB myofibers showed NMJ-centered intracellular FM 1–43 gradient, whereas only 6% ± 2% WT myofibers exhibited this phenomenon (*p* < 0.001). These data substantiate the finding that NMJs are focally more susceptible to injury than other regions of the sarcolemma, and exercise-induced NMJ injury is dramatically exacerbated in the G93A ALS mice.

### 3.4. Mitochondrial Dysfunction Is Associated with the Disruption of Cell Membrane Integrity at NMJs of the ALS Muscle

In skeletal muscle, the cell surface membrane travels deep into the myofibril-forming transverse tubules (T-tubules). Freshly isolated FDB myofibers were incubated with α–BTX, CellMask, and TMRE to locate the NMJs and T-tubule network and to record mitochondrial potentials, respectively (Figure 4D). Normal polarized mitochondria and organized T-tubule networks were observed in WT myofibers (Figure 4D, left). Myofibers derived from the 4-month-old G93A mice showed fully depolarized mitochondria at NMJs (Figure 4D, right), and those derived from the 2-month-old G93A mice showed partially depolarized mitochondria (Figure 4D, middle). Remarkably, disorganized T-tubule networks were observed near the NMJs in 2-month-old G93A myofibers, which became further exacerbated at 4 months, including regional loss of T-tubules (hollow area under the NMJ) and formation of enlarged spheres. Pearson’s chi-squared tests demonstrated a significant correlation between T-tubule disruption and mitochondrial depolarization at the NMJ in both 2-month (*p* < 0.0001) and 4-month (*p* < 0.01) G93A myofibers (Figure 4E). These data confirm that mitochondrial dysfunction is associated with the disruption of cell membrane integrity at NMJs of the ALS muscle.

We and others have shown that mitochondrial dysfunction is associated with enhanced reactive oxygen species (ROS) production in the ALS mouse muscle [1,4,18,46], which could impact the intrinsic membrane repair function of MG53 [47,48]. Using our established live cell imaging method, we examined whether oxidative stress impacts the trafficking of MG53 vesicles inside C2C12 cells overexpressing GFP–MG53 [49]. The 2D x–y time-lapse images were continuously recorded for 10 s in the presence or absence (basal) of 1 mM H_2_O_2_. Representative images at 0 s (0 s, pseudo color green), 5 s (5 s, pseudo color red) and 10 s (10 s, pseudo color red) were selected for generating the overlay images (Figure 4F). The overlay images of 0 s with 5 s or 0 s with 10 s provide a visualization of GFP–MG53 vesicle dynamics. In the overlay images, non-moving vesicles are marked by yellow color (completely overlap), while moving vesicles are indicated by the red and green colors (no overlap). Note that there is almost no detectable movement of GFP–MG53 vesicles in the presence of H_2_O_2_, while moving GFP–MG53 vesicles were detected under basal condition. Together, these data suggest that oxidative stress directly limits the movement of MG53 vesicles, which could directly impair MG53-mediated membrane repair function and contribute to the MG53 degeneration of NMJs and sarcolemma in ALS skeletal muscle.

### 3.5. Impaired MG53 Membrane Repair Function Is a Common Pathological Feature of ALS Muscle

Both sporadic ALS (sALS) and familial ALS (fALS) patients show remarkable mitochondrial defects in motor neuron and skeletal muscle [50,51,52,53,54,55] and in NMJ degeneration [56,57]. We asked whether impaired MG53 function is a common pathological fact in human ALS skeletal muscle. With the support of the Target ALS Human Postmortem Tissue Core, we obtained paraffin-embedded diaphragm and psoas muscle sections from both sporadic and familial ALS decedents and non-ALS controls. The demographics of human decedents and the biospecimen information for human samples are listed in Supplementary Tables S1 and S2. The ten ALS decedents (ages ranging from 34 years old to 72 years old) cover both sALS (eight cases) and fALS (two cases), in which five diaphragm and six psoas samples are available. Three non-ALS muscle samples derived from human decedents at the ages of 22, 52, and 63 years old approximately covered the age range of the ALS decedents. There were two diaphragm and three psoas samples available from those non-ALS controls. Note that the non-ALS controls suffered cancer, but without central nerve system metastasis. We performed MG53 immunostaining on both longitudinal and transverse sections of these human skeletal muscle samples.

**Figure 5:**
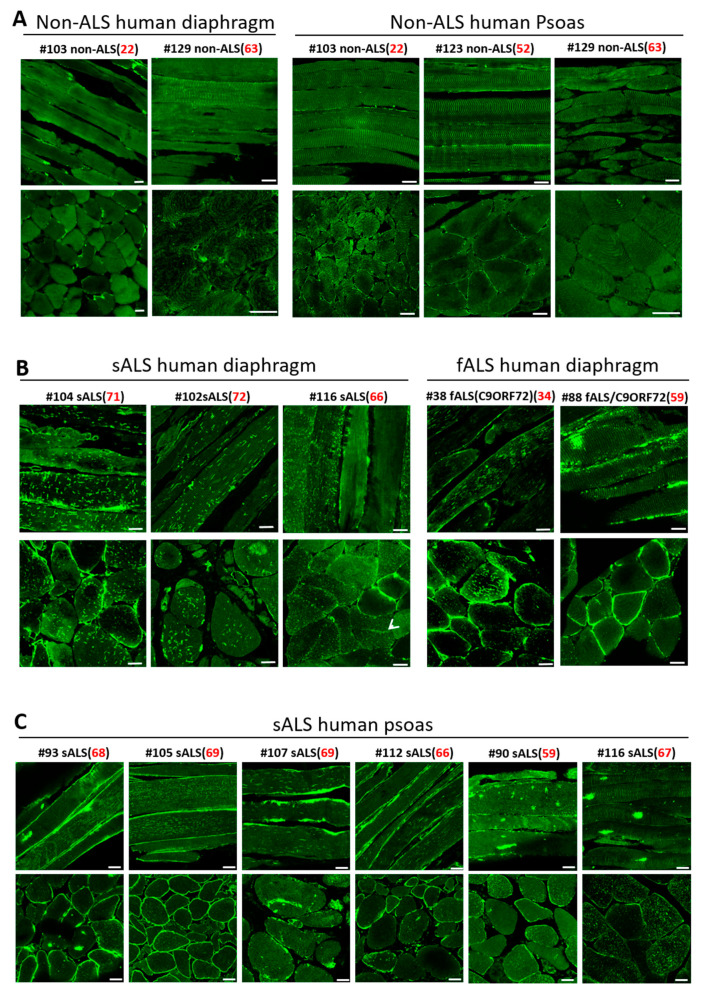
MG53 aggregation is a common pathological feature of skeletal muscle in both sporadic and familial human ALS. (**A**) Longitudinal (top panels) and cross-sectional (bottom panels) staining of MG53 demonstrated uniform patterns with few MG53 aggregates in non-ALS human diaphragm (left) and psoas muscle (right). (**B**) Human ALS diaphragms (sALS: sporadic ALS: fALS: familial ALS) displayed extensive intracellular aggregates of MG53. (**C**) Human ALS psoas muscles derived from both sALS and fALS displayed extensive intracellular MG53 aggregates. The age at the death is indicated in red font. Scale bars: 20 µm.

Both longitudinal and transverse sections of human ALS diaphragm (Figure 5B) and psoas muscle (Figure 5C) showed dramatic intracellular MG53 aggregates and abnormal sarcolemma-targeting pattern. In contrast, the muscle samples of non-neurological control decedents showed more diffused cytosolic MG53 distribution, with only a few scattered MG53 aggregates (Figure 5A). It is not unexpected to see a few MG53 aggregates in non-ALS muscle, as MG53-mediated membrane repair also occurs under normal conditions, but to a much lesser extent. As shown in Figure 2B,C, this same staining pattern was observed in longitudinal and transverse sections of diaphragm, EDL, TA, soleus, and FDB muscles derived from G93A mice. Together, the data collected from both familial and sporadic ALS decedents and from the G93A mouse model suggest that compromised MG53-mediated muscle membrane repair function could be a common pathology in ALS.

### 3.6. Recombinant Human MG53 Protein Preserves Membrane Integrity of Diaphragm in G93A Mice

**Figure 6:**
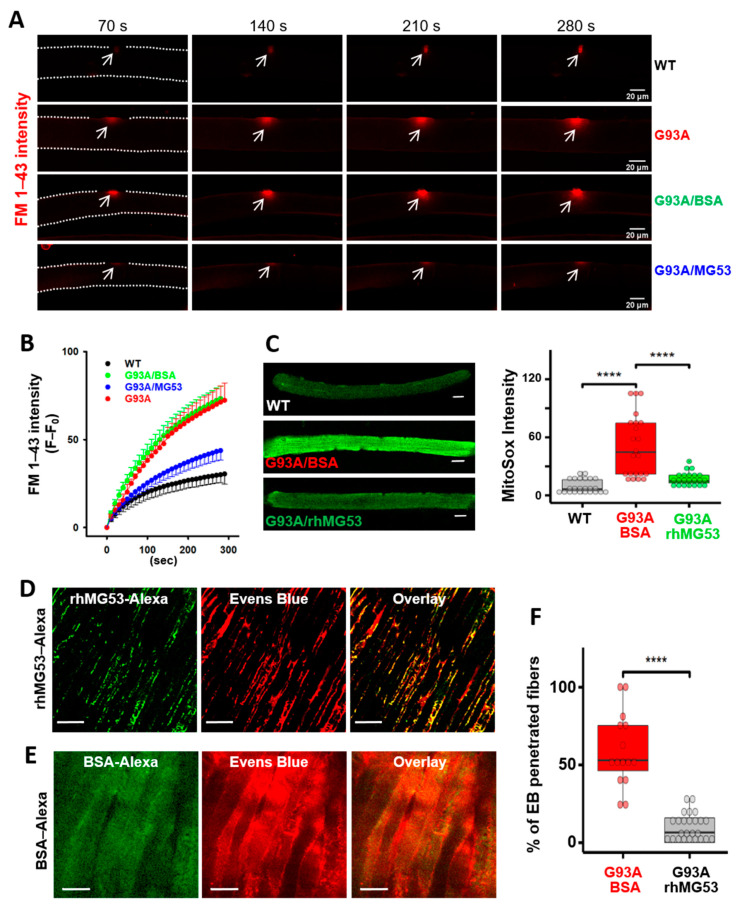
rhMG53 preserved membrane integrity of the diaphragm in G93A mice. (**A**) Time-lapse recordings of FM 1–43 accumulation in FDB myofibers after laser-induced membrane damage. The dashed lines highlight the myofiber outline. (**B**) Plotting of FM 1–43 intensity inside FDB myofibers over time after laser-induced membrane injury. Between G93A and WT (*n* = 6–9 myofibers/group; *p <* 0.001), and between G93A/BSA (9 myofibers) and G93A/rhMG53 (10 myofibers) (*p* < 0.001). (**C**) Live WT and G93A FDB myofibers pre-treated with rhMG53 (2 µg/mL) or BSA (2 µg/mL, as control) were loaded with MitoSox Red for evaluating the mitochondrial superoxide production. rhMG53 treatment significantly reduced mitochondrial superoxide level in G93A FDB myofibers (WT, *n* = 24; G93A + BSA, *n* = 22; G93A + rhMG53, *n* = 22, **** *p* < 0.0001). Scale bars: 20 µm. (**D**) Exercise-induced accumulation of EB in diaphragm derived from G93A mice (2-month-old) was reduced with IV administration of rhMG53–Alexa (2 mg/kg). rhMG53–Alexa targeted to the sarcolemma of the diaphragm. Scale bars: 20 µm. (**E**) Diaphragm derived from G93A mice receiving BSA–Alexa showed extensive accumulation of EB. BSA–Alexa did not target to the sarcolemma. Scale bars: 20 µm. (**F**) Percentage of myofibers with EB penetration was significantly reduced with administration of rhMG53–Alexa (*n* = 14 for BSA––Alexa488, *n* = 25 for rhMG53––Alexa488 from 3 pair of G93A mice, **** *p* < 0.0001).

The recombinant human MG53 (rhMG53) protein has been shown to protect various cell types against membrane disruption when applied to the extracellular environment, and it ameliorates pathology associated with muscular dystrophy [29], acute lung injury [30], myocardial infarction [31], acute kidney injury [32], and ischemic brain damage [33] in animal models. Here we examined whether rhMG53 has beneficial effects in preserving the integrity of the ALS skeletal muscle. Using an established protocol to evaluate the muscle membrane repair function following laser-induced damage [23,45], we conducted in vitro studies to test (1) whether G93A myofibers showed higher laser-induced fragility compared with WT myofibers and (2) whether administration of rhMG53 could improve membrane integrity of G93A myofibers. A small area of the FDB myofiber (12 μm × 12 μm) was exposed to a high intensity UV laser to cause localized cell membrane injury allowing the entry of FM 1–43 dye. As shown in Figure 6A, G93A myofibers (4-month-old) showed more intracellular accumulation of FM 1–43 compared with WT, indicating fragility and impaired cell membrane repair mechanism in G93A myofibers. In the presence of 10 µg/mL of rhMG53 in the culture medium, the G93A myofibers showed a reduced intracellular accumulation of FM 1–43. Application of the same amount of bovine serum albumin (BSA) was ineffective. The time-dependent FM 1–43 accumulation following laser-induced injury is shown in Figure 6B, demonstrating that extracellular application of rhMG53 improved the membrane repair function of the G93A muscle myofibers. In a previous study of ALS G93A mice, we found that G93A myofibers show oxidative stress with enhanced mitochondrial superoxide production [14]. Here we found that the application of rhMG53 (2 µg/mL) in the medium for 12 h significantly reduced the mitochondrial ROS production in G93A myofibers (Figure 6C).

We next tested whether intravenous (IV) administration of 2 mg/kg rhMG53–Alexa (or BSA–Alexa as a control) in the G93A mice, immediately prior to running, attenuates diaphragm membrane injury. The dosage of rhMG53 was determined based on our previous publications with rhMG53 in preservation of membrane integrity in multiple organs [30,32,33]. The G93A mice also received EB injection 16 h earlier before the testing, as described in Figure 1. After running, diaphragm muscles were immediately collected for live cell imaging of Alexa and EB simultaneously. As shown in Figure 6D, mice treated with rhMG53–Alexa showed prominent membrane patches of MG53–Alexa, indicating that rhMG53–Alexa targets injured diaphragm muscles, similar to our previous study with *mdx* mice [29]. In contrast, diaphragm derived from the G93A mice receiving BSA–Alexa showed a diffuse pattern, indicating that BSA–Alexa could not form membrane patches (Figure 6E). Compared with BSA–Alexa treated mice, the proportion of myofibers positive for intracellular accumulation of EB was significantly reduced in the diaphragm muscles from rhMG53–Alexa treated mice (Figure 6F), confirming the efficacy of rhMG53 to preserve diaphragm membrane integrity in vivo.

### 3.7. Systemic Application of rhMG53 Preserves NMJ Integrity and Extends Life Span of G93A Mice

**Figure 7:**
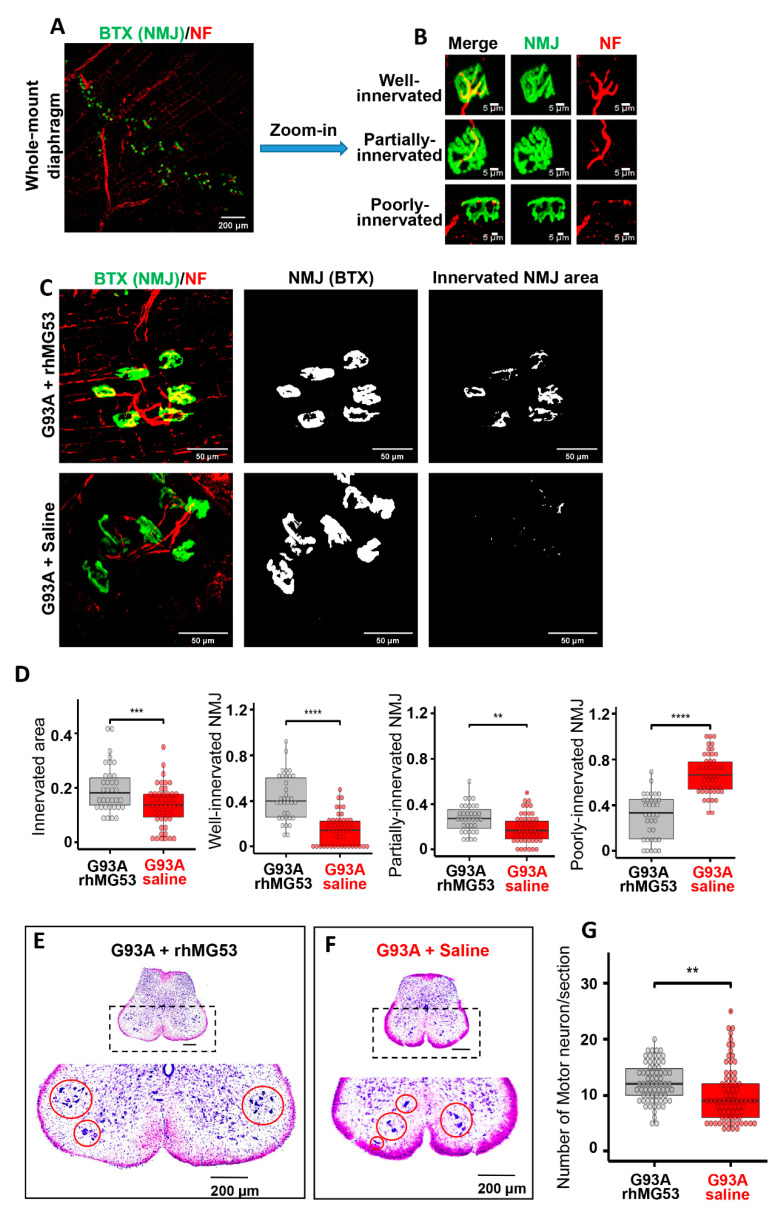
Systemic application of rhMG53 preserved NMJ integrity and promoted motor neuron survival in ALS mice. (**A**) Whole-mount fixed diaphragm muscle stained with the NF antibody (red) for marking axonal terminals and BTX (green) for detecting NMJ. (**B**) Z-stack images of well-innervated (the ratio of innervated area >20%), partially-innervated (the ratio of innervated area between 10% and 20%), and poorly-innervated NMJs (the ratio of innervated area < 10%) in diaphragm muscle. (**C**) Z-stack images of diaphragm muscle from G93A mice receiving 2-week rhMG53 treatment or saline control (left panels). The area of individual NMJ defined by BTX is presented (central panels). The innervated area of NMJ is defined by the area overlapping with NF (right panels). (**D**) Comparing the ratio of innervated NMJ area in rhMG53-treated (*n* = 39, 4 mice) and saline-treated (*n* = 45, 5 mice) diaphragm muscles of G93A littermate mice, as well as the ratio of well-, partially-, poorly-innervated NMJs. rhMG53 treatment significantly preserved the innervation of NMJ in diaphragm muscle of G93A littermate mice. ** *p* < 0.01, *** *p* < 0.001, **** *p* < 0.0001. (**E**,**F**) Images of the lumbar spinal cord section of G93A mice (with 2-weeks of rhMG53 or saline treatment from the age of 3 months). (**G**) The number of surviving motor neurons per section in G93A littermate mice after two-weeks of treatment with rhMG53 (12.2 ± 0.4) or saline (10.1 ± 0.5) (rhMG53, *n* = 70 spinal cord sections; saline, *n* = 77 spinal cord sections; 4 pairs of G93A mice per cohort, ** *p* < 0.01).

Our ultimate goal is to develop rhMG53 as a treatment for ALS, which would be initiated after diagnosis—and thus after symptom onset—to ALS patients. Therefore, we evaluated the efficacy of rhMG53 administration to G93A mice after ALS onset by starting the treatment at the age of 3 months [58]. We adapted a method to quantify NMJ innervation in diaphragm muscle [59] of adult mice. Figure 7A shows a representative image of a mouse diaphragm stained with anti-neurofilament (NF) antibodies (red fluorescence) and BTX (green fluorescence). By changing the focal plane and performing a 3D scan of a zoomed-in area, we could examine NMJ in the entire diaphragm. The 3D scan projection allowed us to distinguish well-innervated NMJs from partially innervated or denervated NMJ (Figure 7B). Ten G93A littermate mice (3-month-old) were divided into two cohorts, one receiving IV injection of rhMG53 (2 mg/kg, daily) and the other receiving saline for 2 weeks. Figure 7C shows representative 3D projection images of the diaphragm muscle from rhMG53- and saline-treated mice. The innervated NMJ area was defined by the overlapping area between BTX and NF signals. Quantitative analyses of NMJ innervation are presented in Figure 7D, demonstrating that rhMG53 treatment could significantly preserve NMJ integrity and maintain well-innervated and partially innervated NMJs, as well as reduce the proportion of denervated NMJs compared with saline-treated controls. In a separate experiment, motor neurons in the anterior horn region (Nissl staining-positive with diameter larger than 25 µm) were counted (Figure 7E,F). The 2-week rhMG53 treatment significantly preserved the number of motor neurons in spinal cords from the G93A mice (rhMG53 vs saline, *p* < 0.01) (Figure 7G), suggesting that this treatment also alleviated motor neuron degeneration.

**Figure 8:**
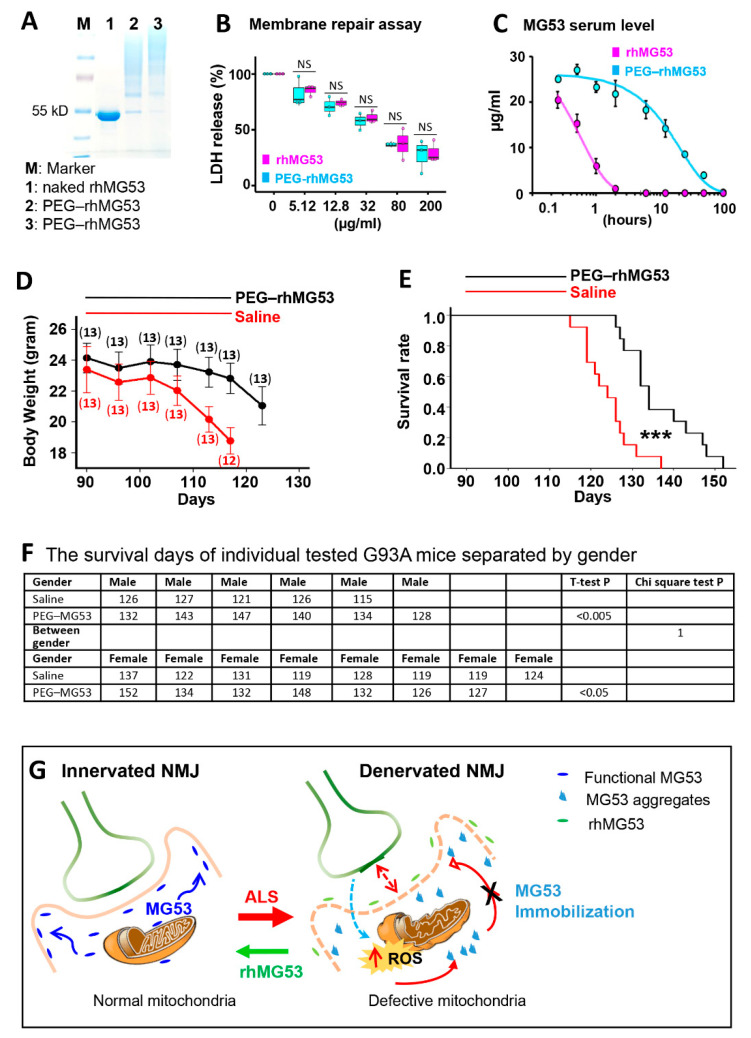
rhMG53 alleviates disease progression and extends the life span of G93A mice. (**A**) PEGylation of rhMG53 is successful based on Coomassie blue staining of the different oligomerization patterns of the protein running on SDS–PAGE. Lanes 2 and 3 represent two independent rhMG53 PEGylation experiments. (**B**) The chemical modification did not affect the membrane repair function of rhM53, as there was no difference in EC_50_ of LDH release from the cultured C2C12 cells following a mechanical membrane damage with glass beads between the rhMG53 and PEG–rhMG53 (*n* = 3 independent experiments). (**C**) Pharmacokinetic (PK) assessment revealed that PEGylation increased serum half-life of rhMG53 in rats from 0.5 h (rhMG53, *n* = 4) to 12 h (PEG–rhMG53, *n* = 3). (**D**) Bodyweight changes of G93A mice receiving PEG–rhMG53 or saline treatment. The numbers of mice included in each data point are indicated on the plot. (**E**) Survival curve of G93A mice with one-month PEG–rhMG53 or saline treatment. The endpoint (death) of a G93A mouse was defined by the loss of righting reflex within 30 s when the mouse was place on its side. (*n* = 13 pairs of G93A littermates, *** *p* < 0.001). (**F**) The survival days of individually tested G93A mice separated by gender. The chi-square test further confirmed that the significant difference between PEG–rhMG53 (PEG–MG53)- and saline-treated groups was independent of gender. (**G**) Proposed mechanisms underlying membrane repair defects at NMJ of ALS.

The short half-life of rhMG53 in circulation (~1 h) may present a hurdle for treating chronic tissue injuries in ALS [32,60]. PEGylation is a well-established method for increasing the half-life of therapeutic proteins in circulation [39,61]. Addition of polyethylene glycol (PEG) often reduces immunogenicity of the PEGylated proteins without a major loss of their biological activity [62,63,64], and several PEGylated proteins have reached the market [64,65]. We produced PEGylated rhMG53 (PEG–rhMG53). As shown in Figure 8A, PEGylation of rhMG53 is successful based on the oligomerization pattern of the protein run on SDS–PAGE. We previously developed an in vitro assay to evaluate the efficacy of rhMG53 in protecting against membrane damage by measuring LDH in the extracellular solution of culture cells, as LDH leaks from the cell into the extracellular solution following cell membrane injury [25,26,29,66]. The PEG-modification did not affect the membrane repair function of rhM53, as the EC_50_ of LDH release did not change (Figure 8B). Meanwhile the half-life of PEG–rhMG53 increased in circulation from 0.5 to 12 h (Figure 8C). This elongated half-life in circulation allowed us to test the PEG–rhMG53 in G93A mice by IV injection on every other day for a longer period.

The PEG–rhMG53 (2 mg/kg, IV) was administered to the G93A mice (3-month-old) every other day for one month. Twenty-six G93A littermate mice (from three litters, with both genders included) were divided into two groups, one receiving PEG–rhMG53, and the other receiving saline as a control. The bodyweights of the PEG–rhMG53 or saline treated mice were recorded. One month of PEG–rhMG53 treatment slowed weight loss compared with the saline-treated G93A mice (Figure 8D). Note that the weight loss of PEG–rhMG53-treated mice accelerated again after the treatment ended. This is not unexpected, as the half-life of PEG–rhMG53 is only 12 h in rodent circulation (Figure 6C). This one-month treatment of PEG–rhMG53 also significantly extended the life span of G93A mice from 124 ± 6 days (saline) to 137 ± 9 days (PEG–rhMG53) (Figure 8E). The endpoint (death) of a G93A mouse was defined by the loss of righting reflex within 30 s when the mouse was place on its side. The survival days of individually treated G93A mice and the statistics for both genders are listed in Figure 8F. While the cohorts were not perfectly gender balanced per litter, the therapeutic benefits of rhMG53 on survival remained when male and female mice were considered separately (survival increased to 137 ± 7 for male mice and 135 ± 10 for females), when compared with the saline-treated G93A littermate mice. The chi-square test further confirmed that the significant difference between PEG–rhMG53- and saline-treated groups was independent of the gender.

## 4. Discussion

ALS patients experience rapid deterioration of skeletal muscle, especially diaphragm wasting that significantly impacts their life span and life quality. We find that muscle membrane damage is a key factor underlying the severe muscle wasting in ALS, a pathological mechanism that has not been explored before. Our study reveals that the membrane at the NMJ is more susceptible to injury than the rest of the sarcolemma and that MG53 forms membrane patches at NMJ following modest exercise training. Thus, MG53 represents an important physiologic component of protection against injury to the NMJ. Due to oxidative stress associated with ALS, the endogenous MG53 gradually becomes dysfunctional and is not sufficient to sustain muscle membrane repair or to maintain NMJ integrity during ALS progression. Systemic administration of exogenous rhMG53 has beneficial effects to restore diaphragm muscle repair and NMJ integrity, leading to increased life span in the ALS mice.

While transient intracellular oxidation initiates MG53 vesicles to form repair-patches [23], exposure of cells to sustained oxidative stress leads to immobilization of MG53′s membrane repair function [47]. It is known that mitochondrial dysfunction in ALS causes intracellular oxidative stress; thus, elevated ROS could impede normal MG53 movement to areas of sarcolemmal damage, resulting in the observed aggregation and loss of its tissue repair function. Abnormal intracellular aggregation of MG53 was observed in multiple types of muscles from the G93A mice. Similarly, the abnormal aggregates were seen in muscle samples from human ALS decedents with both sporadic and familial forms of ALS.

A proposed mechanism underlying the membrane repair defects at the NMJ of ALS is illustrated in Figure 8G. Since NMJ is an active site of neuron–muscle crosstalk, it is conceivable that membrane repair defects in ALS initiate from NMJ. During ALS progression, mitochondrial dysfunction causes elevated ROS production, leading to ectopic MG53 aggregation and disruption of MG53′s tissue repair function, which could exacerbate NMJ denervation. Oxidative stress, muscle membrane damage, and NMJ denervation could form a vicious cycle that promotes muscle wasting and neuronal death in ALS. Future studies are needed to further characterize the detailed molecular mechanism underlying MG53-associated defective sarcolemma repair in ALS muscle and whether other membrane repair proteins are also involved in this process.

We demonstrated that exogenously administered rhMG53 forms repair patches and reduces membrane leakage of the ALS diaphragm muscle. Reducing cell membrane leakage can improve the intracellular milieu and mitigate mitochondrial ROS production, thus improving the intrinsic membrane repair function of MG53 to preserve the integrity of NMJ and muscle membrane. Indeed, systemic administration of rhMG53 or PEG–rhMG53 to the ALS mice after disease onset preserved innervation of the diaphragm and prolonged the lifespan. Studies in mice, rats, and dogs reported no observable toxic effects with long-term administration of rhMG53 [29,32,36]. While we demonstrated that repetitive IV administration of the PEG–rhMG53 has beneficial effects on G93A mice, further studies are required to establish the safety profile of the chemically modified rhMG53 and to test the therapeutic efficacy in other ALS animal models.

Endogenous MG53 is predominantly expressed in striated muscle [23,67] but not in neurons [33]. Under physiological conditions, MG53 in circulation does not cross the brain–blood barrier [33]. Therefore, it was unlikely that rhMG53 in circulation directly reached the motor neuron in spinal cord to protect its function. As ALS appears to be a combination of “dying-forward” and/or “dying-back” pathophysiological processes, and the NMJ is the critical site for this bidirectional crosstalk between motor neurons and myofibers, we speculate that the beneficial effects of rhMG53 on preserving anterior horn motor neuron cell bodies is likely secondary to the preservation of NMJ integrity, which slows the dying-back process of motor neuron degeneration. As the permeability of the brain–blood barrier increases in ALS condition [68,69], contributions from a direct effect on motor neurons remain possible, which will require future exploration.

The effects of exercise training on ALS progression remain controversial [70,71], and diaphragm pacing was found to be associated with reduced survival in ALS patients with respiratory insufficiency [72,73,74,75,76]. Our study with the ALS mice demonstrates that even modest exercise training leads to increased damage to the diaphragm, which may exacerbate ALS progression. This appears to be due to the severely compromised membrane repair capacity of the ALS muscle. Based on this finding, one should be cautious in designing exercise-related protocols or diaphragm pacing as alternative interventions to mitigate ALS. However, it might suggest that exercise regimens and/or diaphragm pacing in the presence of exogenously administered rhMG53 could be beneficial.

## 5. Conclusions

Our study identifies defective muscle membrane repair as a crucial pathological component in ALS. The muscle membrane injury occurs early in the course of ALS and contributes to disruption of NMJ and muscle wasting. Probing the role of mitochondrial ROS production in modulating MG53-mediated cell membrane repair may have broader implications in understanding the basic pathophysiology of ALS. Our study provides proof-of-concept data supporting the beneficial effects of rhMG53 in preserving the integrity of NMJ and muscle cell membrane to alleviate ALS progression.

## Data Availability

All the data are available within the article or Supplementary Materials.

## Supplementary Materials

The following are available online at https://www.mdpi.com/article/10.3390/antiox10101522/s1, Supplementary Figure S1: Additional Western blot data for Figure 2D, Supplementary Figure S2: Western blot results for MG53 in TA muscles derived from G93A and WT mice at the age of 2 months and 4 months, Supplementary Table S1: Demographics of human decedents, Supplementary Table S2: Biospecimen information for human samples.
