# Supplementary material for: MG53 Preserves Neuromuscular Junction Integrity and Alleviates ALS Disease Progression
Source: Antioxidants (Basel). 2021 Sep 25;10(10):1522. doi: 10.3390/antiox10101522 (PMC8532806; doi:10.3390/antiox10101522)
Supplement: Supplementary file 1 [file antioxidants-10-01522-s001.zip › antioxidants-1359724-supplementary3.pdf]

# MG53 preserves neuromuscular junction integrity and alleviates ALS disease progression

JianxunYi[1,2], AngLi[1,2], XuejunLi[1,2], KiHoPark[3], XinyuZhou[3], FrankYi[3], YajuanXiao[2], DosukYoon[2], TaoTan[3], LyleW.Ostrow[4], JianjieMa[3] andJingsongZhou[1,2]

[1]University of Texas at Arlington
[2]Kansas City University of Medicine and Biosciences
[3]The Ohio State University
[4]Johns Hopkins University School of Medicine

¶
Corresponding authors:

Jingsong Zhou
University of Texas at Arlington
Department of Kinesiology
Email: Jingsong.zhou@uta.edu

Jianjie Ma
The Ohio State University
Department of Surgery
Email: Jianjie.ma@osumc.edu

## Supplementary Figure S1

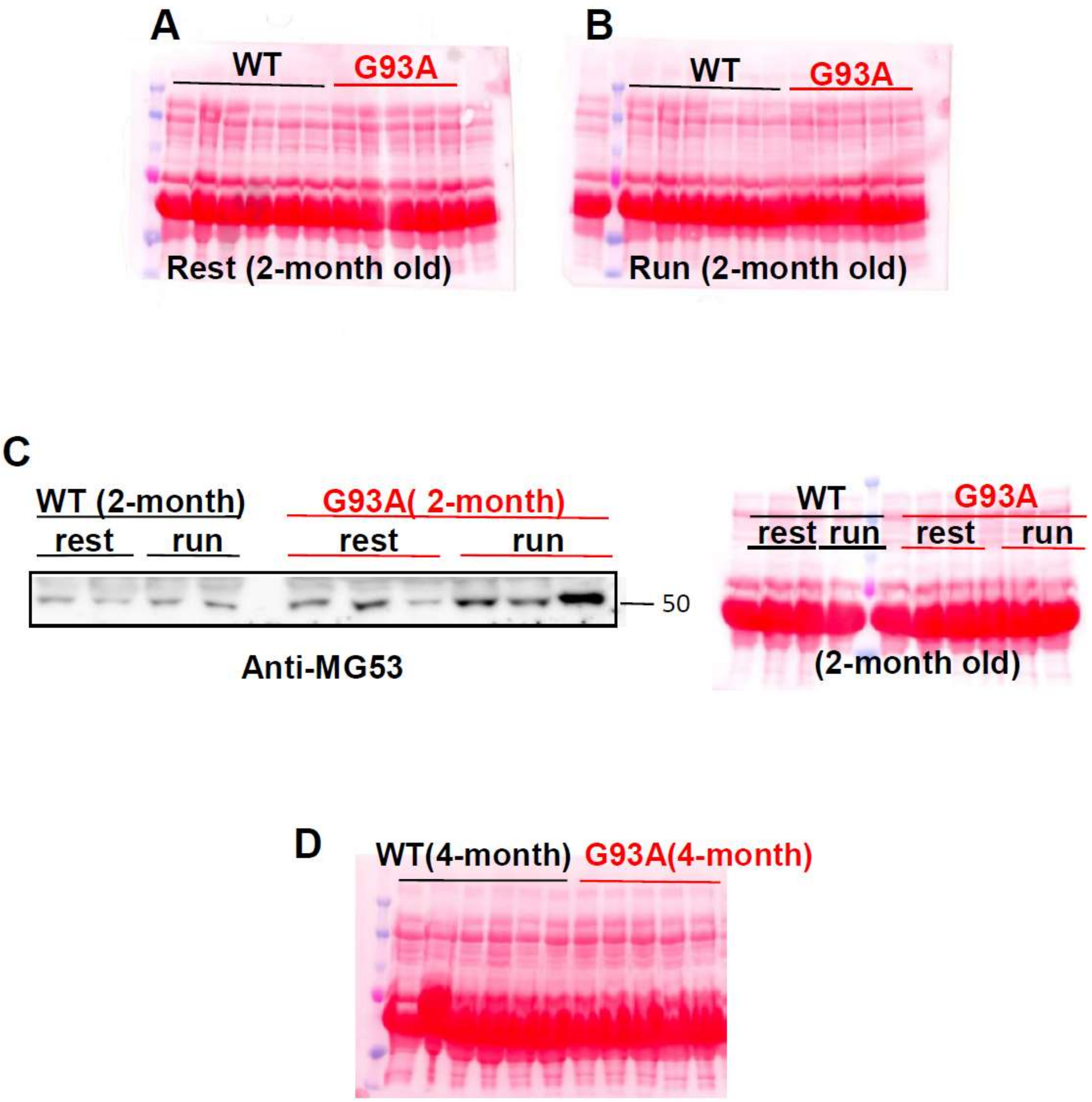


**Supplementary Figure S1** (**A,B**) Ponceau S staining of the immunoblotting assay in **Figure 2D** verifies equal loading amount of the serum samples of 2-month old mice. (**C**) Due to limited numbers of lanes in the acrylamide gel, the additional blotting assay of 2 WT and 3 G93A serum samples before and after running were evaluated in a separate gel. Data from this blot were also included in **Figure 2D** quantification results. (**D**) Ponceau S staining of the immunoblotting assay in **Figure 2E** verifies equal loading amount of the serum samples of 4-month old mice.

## Supplementary Figure S2

A

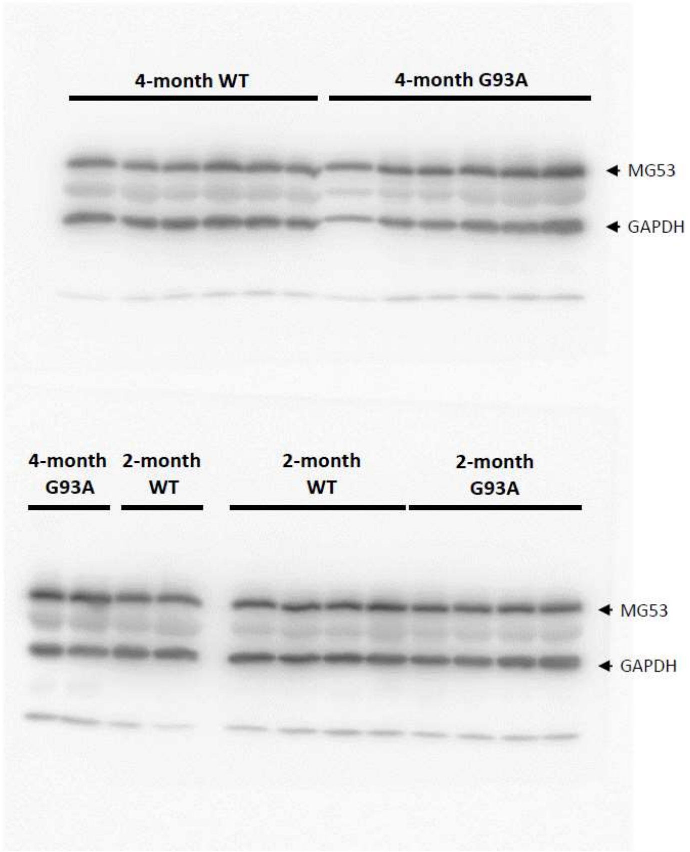


B

| 4 month | | | WT vs G93A: 4M | | 2 month | | | WT vs G93A: 2M |
|---|---|---|---|---|---|---|---|---|
| WT | MG53/GAPDH | mean | p value | | WT | MG53/GAPDH | mean | p value |
| 1 | 0.879 | 0.867 | 0.000210 | | 1 | 0.958 | 1.003 | 0.650967 |
| 2 | 0.809 | | | | 2 | 0.884 | | |
| 3 | 0.778 | | | | 3 | 0.940 | | |
| 4 | 0.912 | | | | 4 | 1.101 | | |
| 5 | 0.860 | | | | 5 | 0.991 | | |
| 6 | 0.962 | | | | 6 | 1.142 | | |
| | | | | | | | | |
| G93A | | | | | G93A | | | |
| 1 | 2.011 | 1.449 | | | 1 | 1.017 | 0.976 | |
| 2 | 1.334 | | | | 2 | 1.047 | | |
| 3 | 1.367 | | | | 3 | 0.924 | | |
| 4 | 1.157 | | | | 4 | 0.915 | | |
| 5 | 1.395 | | | | | | | |
| 6 | 1.329 | | | | | | | |
| 7 | 1.354 | | | | | | | |
| 8 | 1.647 | | | | | | | |

**Supplementary Figure S2.** (**A**) The original blots for MG53 protein level in TA muscles derived from G93A and WT mice at the age of 2-month and 4-month. Anti-MG53 and anti-GAPDH were applied together during blotting. (**B**) Quantification of the MG53 expression level normalized to the GAPDH expression. Note, there in no significant changes in MG53 expression level at the age of 2 months, p>0.5, n=4-6 mice; while the MG53 expression level is significantly higher in G93A muscle at the age of 4 months, p=0.0002, n=6-8 mice.

**Supplementary Table S1**: Demographics of humandecedents

| ID | Race/ Gender | Ageat onset | Ageat death | Site of onset | Diagnosis | C9ORF72 expansion | SOD1 mutation |
|---|---|---|---|---|---|---|---|
| 38 | WF | 33 | 34 | Upper + lower extremities | fALS | Yes | No |
| 88 | WM | 57 | 59 | Bulbar | fALS + FTD | Yes | No |
| 90 | WF | 57 | 59 | Bulbar + Respiratory | sALS | No | No |
| 93 | WM | 65 | 68 | left hand | sALS | No | No |
| 102 | BF | 70 | 72 | Bulbar | sALS | No | No |
| 104 | WF | 69 | 71 | Bulbar | sALS | No | No |
| 105 | WM | 61 | 69 | left leg | sALS | No | No |
| 107 | WM | 67 | 69 | left hand | sALS | No | No |
| 112 | WM | 67 | 69 | right hand | sALS | No | No |
| 116 | WF | 64 | 67 | Left foot | sALS | No | No |

| ID | Race/ Gender | Age at death | Diagnosis | Cause of Death |
|---|---|---|---|---|
| 103 | WM | 22 | Non-Neurological Control | Metastatic synovial sarcoma (no CNS mets) |
| 123 | BM | 52 | Non-Neurological Control | Pancreatic Cancer (no CNS mets) |
| 129 | WF | 63 | Non-Neurological Control | Colon cancer with liver & lymph node mets (no CNS mets) |

**Supplementary Table S2**: Biospecimen information for humansamples

| ID | Biospecimen type | Anatomic site | Vitalstate ofpatient | Collection | Type of stabilization | Type oflong term preservation | Constitution of preservative | Storage duration | Shipping temperature | Composition Assessment &selection |
|---|---|---|---|---|---|---|---|---|---|---|
| 38 | Skeletal muscle | Diaphragm | Postmortem | Autopsy | Formalin fixed | Paraffin embedded | 10% neutral-buffered formalin | 21years | 25°C | Clinical, genetic & neuropathological |
| 88 | Skeletal muscle | Diaphragm | Postmortem | Autopsy | Formalin fixed | Paraffin embedded | 10% neutral-buffered formalin | 1-7years | 25°C | Clinical, genetic & neuropathological |
| 90 | Skeletal muscle | Psoas | Postmortem | Autopsy | Formalin fixed | Paraffin embedded | 10% neutral-buffered formalin | 1-7years | 25°C | Clinical, genetic & neuropathological |
| 93 | Skeletal muscle | Psoas | Postmortem | Autopsy | Formalin fixed | Paraffin embedded | 10% neutral-buffered formalin | 1-7years | 25°C | Clinical, genetic & neuropathological |
| 102 | Skeletal muscle | Diaphragm | Postmortem | Autopsy | Formalin fixed | Paraffin embedded | 10% neutral-buffered formalin | 1-7years | 25°C | Clinical, genetic & neuropathological |
| 104 | Skeletal muscle | Diaphragm | Postmortem | Autopsy | Formalin fixed | Paraffin embedded | 10% neutral-buffered formalin | 1-7years | 25°C | Clinical, genetic & neuropathological |
| 105 | Skeletal muscle | Psoas | Postmortem | Autopsy | Formalin fixed | Paraffin embedded | 10% neutral-buffered formalin | 1-7years | 25°C | Clinical, genetic & neuropathological |
| 107 | Skeletal muscle | Psoas | Postmortem | Autopsy | Formalin fixed | Paraffin embedded | 10% neutral-buffered formalin | 1-7years | 25°C | Clinical, genetic & neuropathological |
| 112 | Skeletal muscle | Psoas | Postmortem | Autopsy | Formalin fixed | Paraffin embedded | 10% neutral-buffered formalin | 1-7years | 25°C | Clinical, genetic & neuropathological |
| 116 | Skeletal muscle | Diaphragm/ psoas | Postmortem | Autopsy | Formalin fixed | Paraffin embedded | 10% neutral-buffered formalin | 1-7years | 25°C | Clinical, genetic & neuropathological |
| | | | | | | | | | | |
| 103 | Skeletal muscle | Diaphragm/ psoas | Postmortem | Autopsy | Formalin fixed | Paraffin embedded | 10% neutral-buffered formalin | 1-7years | 25°C | Clinical, genetic & neuropathological |
| 123 | Skeletal muscle | Psoas | Postmortem | Autopsy | Formalin fixed | Paraffin embedded | 10% neutral-buffered formalin | 1-7years | 25°C | Clinical, genetic & neuropathological |
| 129 | Skeletal muscle | Psoas | Postmortem | Autopsy | Formalin fixed | Paraffin embedded | 10% neutral-buffered formalin | 1-7years | 25°C | Clinical, genetic & neuropathological |
